# Economic evaluation of the breast cancer screening programme in the Basque Country: retrospective cost-effectiveness and budget impact analysis

**DOI:** 10.1186/s12885-016-2386-y

**Published:** 2016-06-01

**Authors:** Arantzazu Arrospide, Montserrat Rue, Nicolien T. van Ravesteyn, Merce Comas, Myriam Soto-Gordoa, Garbiñe Sarriugarte, Javier Mar

**Affiliations:** Gipuzkoa AP-OSI Research Unit, Integrated Health Organization Alto Deba, Avda Navarra 16, 20500 Arrasate-Mondragón, Gipuzkoa Spain; Aging and Chronicity Health Services Research Group, BIODONOSTIA Research Institute, Paseo Dr Beguiristain s/n, 20014 Donostia, Gipuzkoa Spain; REDISSEC (Red de Investigación en Servicios de Salud en Enfermedades Crónicas – Spanish Health Services Research on Chronic Patients Network), Bilbao, Bizkaia Spain; Basic Medical Sciences department, Biomedical Research Institute of Lleida, University of Lleida, Avda. Rovira Roure 80, 25198 Lleida, Spain; Department of Public Health, Erasmus University Medical Center Rotterdam, Dr Molewaterplein 50, 3015 GE Rotterdam, The Netherlands; Evaluation and Epidemiology Department, Hospital del Mar – IMIM (Hospital del Mar Medical Research Institute), Passeig Maritim 25-29, 08003 Barcelona, Spain; Breast Cancer Early Detection Programme, Public Health Division of Bizkaia, Basque Government, Alameda Rekalde 39, 48008 Bilbao, Bizkaia Spain; Health Management Service, Integrated Health Organization Alto Deba, Avda Navarra 16, 20500 Arrasate-Mondragón, Gipuzkoa Spain

**Keywords:** Breast cancer, Screening, Cost-effectiveness, Budget impact analysis, Simulation, Modelling, Evaluation, Public health

## Abstract

**Background:**

Breast cancer screening in the Basque Country has shown 20 % reduction of the number of BC deaths and an acceptable overdiagnosis level (4 % of screen detected BC). The aim of this study was to evaluate the breast cancer early detection programme in the Basque Country in terms of retrospective cost-effectiveness and budget impact from 1996 to 2011.

**Methods:**

A discrete event simulation model was built to reproduce the natural history of breast cancer (BC). We estimated for lifetime follow-up the total cost of BC (screening, diagnosis and treatment), as well as quality-adjusted life years (QALY), for women invited to participate in the evaluated programme during the 15-year period in the actual screening scenario and in a hypothetical unscreened scenario. An incremental cost-effectiveness ratio was calculated with the use of aggregated costs. Besides, annual costs were considered for budget impact analysis. Both population level and single-cohort analysis were performed. A probabilistic sensitivity analysis was applied to assess the impact of parameters uncertainty.

**Results:**

The actual screening programme involved a cost of 1,127 million euros and provided 6.7 million QALYs over the lifetime of the target population, resulting in a gain of 8,666 QALYs for an additional cost of 36.4 million euros, compared with the unscreened scenario. Thus, the incremental cost-effectiveness ratio was 4,214€/QALY. All the model runs in the probabilistic sensitivity analysis resulted in an incremental cost-effectiveness ratio lower than 10,000€/QALY. The screening programme involved an increase of the annual budget of the Basque Health Service by 5.2 million euros from year 2000 onwards.

**Conclusions:**

The BC screening programme in the Basque Country proved to be cost-effective during the evaluated period and determined an affordable budget impact. These results confirm the epidemiological benefits related to the centralised screening system and support the continuation of the programme.

**Electronic supplementary material:**

The online version of this article (doi:10.1186/s12885-016-2386-y) contains supplementary material, which is available to authorized users.

## Background

The evaluation of breast cancer (BC) screening is the subject of a controversial debate regarding its benefit and harms [1, 2]. The BC Screening Programme in the Basque Country (BCSPBC) invited more than 400,000 women from its start in 1996 through 2011 involving more than 1.3 million mammograms. Therefore a great annual investment was assigned in order to obtain future health benefit. During this period (1996–2011) the screening programme reduced 20 % the number of BC deaths whereas 4 % of screen detected BC were over-diagnosed, which has been found to be an acceptable level [1, 3]. Although, these figures support the continuity of the programme, such a mass preventive intervention must be evaluated also in economic terms to warrant that the allocated resources are a worthwhile investment for the entire population [4].

As BC screening has been employed differently throughout the world [5], its evaluation needs to be fitted to the features of the actual women screened and to the implementation of the programme in reality. It is necessary to adopt a population-based approach in order to reflect all the demographic, epidemiological and clinical characteristics of the target population. In contrast with single cohort models, population-based models allow taking into account the heterogeneous composition of the population [6]. At the same time, this approach involves modelling the costs and benefits of all patients comprising both the cohort starting screening in the current year and those already undergoing screening from previous years [7]. Moreover, the interaction of population dynamics and heterogeneity, specially related to aging, could have a substantial effect on the final result of the evaluation [6, 8]. Although Markov modelling is the most common approach in cost-effectiveness analysis, discrete-event simulation models permit more flexible structures which allows including all these characteristics in a single model [9, 10]. Using discrete-event simulation an artificial entity is created for each woman included in the BCSPBC and it is permitted to assign all kind of attributes to this entity in order to specify the evolution of that woman related to breast cancer and the correspondent effect of screening. By including the whole amount of entities that individually represent the invited women, the target population can be reproduced. Allowing multi-cohort modelling is a key advantage of discrete-event simulation in order to carry out economic evaluation of public health programmes.

In the context of the BCSPBC, we can retrospectively examine the cost and effectiveness for the period 1996 through 2011. Recently, a simulation model was developed with the aim of estimating the effect of the BCSPBC mainly in terms of BC mortality decrease and overdiagnosed cases [3]. We have used the same model, already calibrated and validated, to estimate overall costs and quality adjusted life years (QALY) attributable to the screening programme. Additional information in terms of budget impact analysis will help decision-makers to fully understand the economic impact of the screening programme on the budget of the Basque health system. Cost-effectiveness analysis and budget impact analysis provide complementary information and both are necessary when a large volume of the population is involved in the assessed intervention [11].

The aim of this study was to carry out the evaluation of the BC early detection programme in the Basque Country in terms of cost-effectiveness and budget impact from 1996 to 2011.

## Methods

A discrete event simulation model [9, 10] was built to reproduce the natural history of BC according to the key characteristics of the female population invited into the programme from its beginning in 1996 through 2011 [3]. The screening test for BCSPBC consisted of mammography with double projection carried out biennially on all women aged 50 to 69 years. The target population comprised multiple cohorts of women; not only women who were invited to the programme for the first time but also successive invitations for those already included in the BCSPBC [7, 12], thus a multiple-cohort model (dynamic model) was used to represent the whole population including women invited in different calendar years. The model allowed lifetime follow-up for each woman invited to the programme to measure both the long-term costs and benefits of screening. The evaluation period was defined as 1996 through December 31, 2011, as the target population of the programme was changed during 2012 and extended to women in their 40’s with a first-degree family history of BC. However, the simulation model allowed lifetime follow-up in order to estimate the future effects of the screening during the evaluated period. The Ethics Committee for Clinical Research in Gipuzkoa Health Area evaluated and approved the study.

### Model overview

We modelled the natural history of BC using the approach of Lee et al. [13]. Four main states of health were distinguished: (1) disease-free or undetectable BC; (2) asymptomatic BC that could be diagnosed by screening; (3) symptomatic BC diagnosed clinically; and (4) death from BC. Time-to-event distributions used for the modelling of the natural history of BC were obtained from previous studies [13–15]. All-cause mortality, excluding breast cancer specific mortality was also included as a competing risk [16].

Other model input data, such as the exact number of women invited for the first time and their age at the first invitation, programme sensitivity and specificity, the number of positive mammography results and the additional diagnostic tests carried out, and age- and stage-specific cancer incidence were obtained from the BCSPBC database. The final model was calibrated to obtain the closest possible results to observed data. A full description of the model has already been published [3], however a Methodology Appendix (Additional file 1) which describes the main model details and contains a simplified diagram of the model is also available online.

### Utilities

Due to the lack of quality of life estimations in women affected by BC we decided to apply the methodology described by Stout et al to estimate the age-specific quality-of-life utility weights for the different health states [17]. The first step consisted of obtaining age-specific EuroQol EQ-5D quality-of-life utility weights for general Spanish women population [18]. Following the aforementioned approach, specific percentages were applied to general population utilities in order to estimate the potential negative effects of a BC diagnosis during the first year of treatment and end of life (Table 1). We considered end of life equivalent to the metastatic stage in terms of quality of life and duration.

**Table 1:** Quality of life weights in Spanish women population and its reduction due to breast cancer detection

	Health state			
Age	Healthy [18]	In Situ or Stage I	Stage II or III	Stage IV
50–64	0.824	0.742	0.618	0.495
65–74	0.770	0.693	0.578	0.462
75–84	0.682	0.614	0.512	0.409
>84	0.563	0.507	0.422	0.338

### Costs

The perspective of the Basque National Health Service was considered for the economic evaluation. We included both BC diagnosis (screening and additional diagnosis tests) and treatment costs (initial, follow-up and end of life), based on resource consumption and unit costs of the Basque Health Services. The methodology of calculating the unitary costs is fully described elsewhere by Arrospide et al. [19].

The diagnostic costs included screening mammography (42.28€) and other diagnostic tests carried out in the reference hospital such as echography (44.14€), fine needle aspiration (113.49€), core needle biopsy (127.46€) and surgical biopsy (2,594€). Attendants were classified in 5 groups according to screening mammography evidence for BC. Women in the highest groups (3 to 5) were assigned additional tests, one or several, according to the probability observed in the programme data base for the correspondent evidence group.

Treatment costs for BC detected in a clinical stage other than IV were divided into initial and 5-year follow-up costs. When BC was the cause of death, we incorporated the increased costs of the last year of life using the cost of metastatic stage. Initial treatment costs included surgery, radiotherapy and chemotherapy. Pharmacological treatment and medical consultations were incorporated in follow-up costs. For cases of metastatic BC, only annual follow-up costs were calculated. The initial cost was 9.838€ for stage 0, 17.273 for I, 22.145 for II, and 28.776 for III. The follow-up annual cost was 172€ for stage 0, 908 for 1,994 for II, and 1,166 for III. The annual cost for stage IV was 17,879€.

### Cost-effectiveness analysis

Two identical populations were created and followed until death to estimate lifetime costs and QALYs in the screened and unscreened populations. Women in the screened arm were invited according to BCSPBC implementation and no screening mammography was simulated from year 2011 onwards. However, lifetime time horizon was applied to the model to include long-term screening effects. According to the approach applied by Stout et al, during this 15-year period (retrospective time), neither costs nor QALYs were discounted, and a 3 % annual discount rate was applied prospectively to both costs and QALYs, beginning from the end of the evaluated period (31st December 2011) until death [17, 20]. In addition, a complementary scenario with no discount (0 % discount) applied was also considered.

The same model was employed to calculate the ICER for the case of a single cohort of 50,000 women aged 50 years invited to join the programme for the first time in 1996. We used the same alternatives as in the population level approach (with and without screening). As cost-effectiveness analysis is generally applied for a single cohort, these complementary results permit comparison with published data.

### Probabilistic sensitivity analysis

**Fig. 1:**
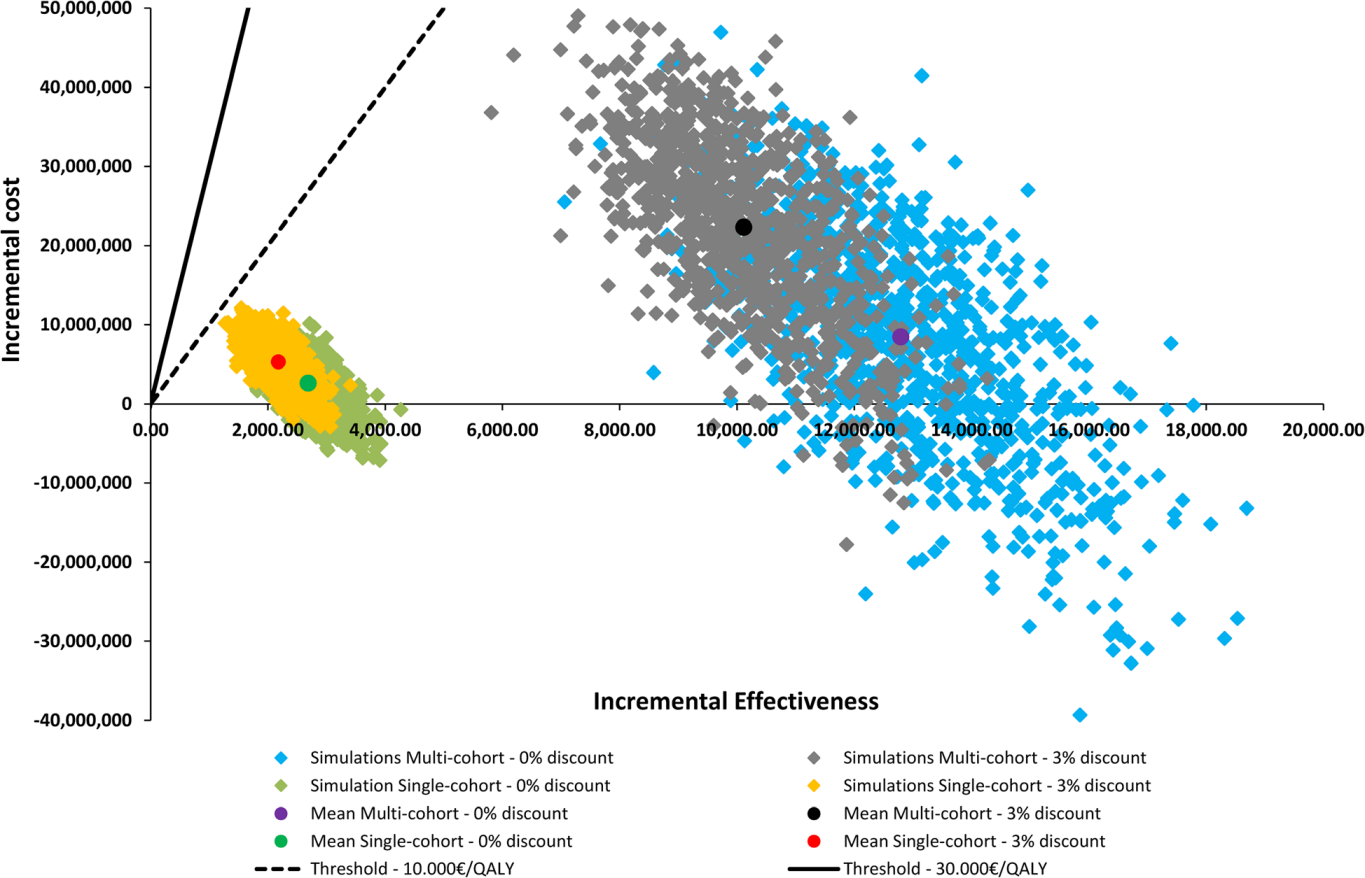
Short title: Cost-effectiveness plane for the period from 1996 through 2011. Detailed legend: Cost-effectiveness plane showing the variability in population-level cost-effectiveness analysis for the period from 1996 through 2011

The probabilistic feature of the model was based on varying the main variables randomly at the same time [21]. Each variable was assigned a distribution fitting the range of all possible values and at the beginning of each simulation a random generator selected the value for each variable from the specified distribution. This permitted to examine the effect of joint uncertainty in the variables of the model through cost-effectiveness plane and acceptability curve [21]. The cost-effectiveness plane displays the incremental cost (vertical axis) and effectiveness (horizontal axis) results of 1,000 simulation runs (Fig. 1). The mean value and 95 % confidence intervals (CI) were shown for the total costs and QALYs, for the differences between the results for the two scenarios, and for the ICER. The distributions used for the main parameters varied in the probabilistic sensitivity analysis were detailed in the Methodology Appendix (Additional file 1).

Variability in participation rates was not included in the main probabilistic sensitivity analysis as variability was assumed very small. However, as we were concerned about the interest on the variation of this parameter we ran cost-effectiveness analysis for the main single-cohort model in two more scenarios with lower participation rates: 50 and 30 %.

### Budget impact analysis

The simulation model built for multi-cohort cost-effectiveness analysis was used simultaneously for budget impact analysis. Cost-effectiveness analysis allows estimating the additional benefit of a new treatment in relationship with its cost and permit comparing the results to those obtained for already accepted treatments. Undoubtedly, the framework described for cost-effectiveness analysis is accepted by experts panels all over the world [8, 22]. However there are some doubts about its real application when health services management is based on a fixed budget. Budget impact analysis provides a new tool to estimate the effect of the decision hold on the future budget of the health services. As defined by Mauskopf et al. budget impact analysis assesses the impact of a new intervention in annual costs, annual health benefits and other important outcomes from its implementation onwards [11, 23].

The model was developed to calculate the annual costs for BC diagnosis and treatment in both the screened and unscreened populations. Diagnostic resources included screening or symptomatic mammograms, as well as other additional diagnostic tests that were implemented in the reference hospital. Treatment costs involved the initial treatment of the BC detected each year and follow-up therapy for prevalent BC, as well as end-of-life costs for those who died from BC. As the budget impact analysis presented financial streams over time, it was not necessary to discount the costs [11].

## Results

The results of the population-level cost-effectiveness analysis are shown in Table 2. The 15-year evaluation demonstrated a cost of 1,126.6 million euros (1,608.7 million euros, undiscounted) and a provision of 6.70 million QALYs (8.84 million QALYs, undiscounted) for lifetime follow-up. In the non-screened scenario, these values were reduced to 1,090.2 million euros and 6.69 million QALYs. Thus, the ICER was 4,214€ per QALY (2,294€/QALY, undiscounted). When disaggregated costs are analysed, 92 % of the total costs were attributed to BC treatment in the screened population. Over the entire study period more than 55 million euros were invested in BC screening mammography, with an additional 12 million for further diagnostic tests, whereas only four million euros were saved in clinical or symptomatic diagnosis. Early detection also involved a savings of more than 27 million euros in the treatment of BC detected in the evaluated population. When a usual single-cohort cost-effectiveness analysis was carried out, the final results were similar in terms of ICER (Table 3).

**Table 2:** Cost-effectiveness analysis of breast cancer screening using the multi-cohort (population level) approach

	0 % discount^a^			3 % discount^a^		
	Mean	95 % CI		Mean	95 % CI	
Screened population						
Total costs (Million Euros)	1,608.7	1,566.0	1,651.7	1,126.6	1,097.8	1,155.3
Screening mammography costs	55.3	55.2	55.5	55.3	55.2	55.5
Screening diagnosis workup	12.1	11.5	12.7	12.1	11.5	12.7
Clinical cancers diagnosis workup	26.1	25.2	27.0	18.3	17.6	18.9
Treatment costs	1,515.1	1,472.8	1,557.5	1,040.9	1,012.5	1,069.3
QALYs	8,845,493	8,828,791	8,862,195	6,696,959	6,684,899	6,709,019
Unscreened population						
Total costs (Million Euros)	1,584.3	1,538.8	1,629.8	1,090.2	1,059.2	1,121.3
Screening mammography costs	0.00	0.00	0.00	0.0	0.0	0.0
Screening diagnosis workup	0.00	0.00	0.00	0.0	0.0	0.0
Clinical cancers diagnosis workup	30.2	29.2	31.11	22.2	21.5	22.9
Treatment costs	1,554.1	1,509.0	1,599.24	1,068.0	1,037.3	1,098.8
QALYs	8,834,785	8,818,066	8,851,504	6,688,293	6,676,240	6,700,347
Difference (Screened - Unscreened)						
Total costs (Million Euros)	24.4	8.5	40.3	36.4	24.6	1,557.5
Screening mammography costs	55.3	55.2	55.5	55.3	55.2	55.5
Screening diagnosis workup	12.1	11.5	12.7	12.1	11.5	12.7
Clinical cancers diagnosis workup	−4.0	−5.1	−2.9	−3.9	−4.8	−3.1
Treatment costs	−39.0	−54.8	−23.1	−27.1	−38.9	−15.4
QALYs	10,708	9,499	11,917	8,666	7,746	9,586
ICER	2,294	738	3,850	4,214	2,703.41	5,725

*CI* confidence interval, *QALY* quality-adjusted life years, *ICER* incremental cost-effectiveness ratio

^a^Discount applied beginning from the end of the evaluated period until death

**Table 3:** Cost-effectiveness analysis of breast cancer screening using a single cohort

	0 % discount^a^			3 % discount^a^		
	Mean	95 % CI		Mean	95 % CI	
Screened population						
Total costs (Million Euros)	213.0	204.7	221.3	161.9	155.9	167.8
Screening mammography costs	12.5	12.458	12.5	12.5	12.5	12.5
Screening diagnosis workup	2.9	2.7	3.1	2.9	2.8	3.1
Clinical cancers diagnosis workup	3.0	2.9	3.2	2.2	2.1	2.3
Treatment costs	194.5	186.3	202.8	144.2	138.3	150.1
QALYs	1,231,858	1,228,748	1,234,968	997,681	995,195	1,000,168
Non-screened population						
Total costs (Million Euros)	206.7	197.4	216.0	153.2	146.5	160.0
Screening mammography costs	0.0	0.0	0.0	0.0	0.0	0.0
Screening diagnosis workup	0.0	0.0	0.0	0.0	0.0	0.0
Clinical cancers diagnosis workup	3.9	3.7	4.1	3.1	2.9	3.2
Treatment costs	202.8	193.6	212.1	150.2	143.5	156.9
QALYs	1,229,578	1,226,441	1,232,715	995,803	993,304	998,301
Difference (Screened - Unscreened)						
Total costs (Million Euros)	6.3	2.5	10.1	8.6	5.7	202.8
Screening mammography costs	12.5	12.5	12.5	12.5	12.5	12.5
Screening diagnosis workup	2.9	2.7	3.1	2.9	2.8	3.1
Clinical cancers diagnosis workup	−0.9	−1.1	−0.7	−0.9	−1.0	−0.7
Treatment costs	−8.3	−12.1	−4.5	−6.0	−8.9	−3.0
QALYs	2,280	1,986	2,575	1,879	1,650	2,108
ICER	2,778	974	4,582	4,623	2,830	6,416

*CI* confidence interval, *QALY* quality-adjusted life years, *ICER* incremental cost-effectiveness ratio

^a^Discount applied beginning from the end of the evaluated period until death

**Table 4:** Cost-effectiveness analysis for a single cohort in different attendance rate scenarios

Participation rate	Incremental costs (Million Euros)	Incremental effectivenes (QALYs)	ICER
0 % discount			
Base Case	6.3	2,280	2,778
50 % attendance	3.2	1,715	1,888
30 % attendance	1.7	1,136	1,453
3 % discount^a^			
Base Case	8.6	1,879	4,623
50 % attendance	5.1	1,409	3,601
30 % attendance	2.9	934	3,051

*QALY* quality-adjusted life years, *ICER* incremental cost-effectiveness ratio

^a^Discount applied beginning from the end of the evaluated period until death

Incremental costs and incremental effectiveness in each of the 1,000 simulations carried out in probabilistic sensitivity analysis are shown graphically in Fig. 1. All the simulations resulted in an ICER lower than 10,000€ per QALY. In addition, the related acceptability curve (Methodology Appendix) showed that in 3 % of the simulations screening was dominant (saved costs) both for the single-cohort and multiple-cohort models when no discount was applied. However, this percentage increased up to 21 % for the single-cohort model and 27 % with population level approach when costs and QALYs were discounted (3 % discount). On the other hand, incremental costs and effectiveness proportionally decreased when lower participation rates were applied in the single-cohort model, therefore the incremental cost-effectiveness ratio result similar in the three scenarios (Table 4).

**Fig. 2:**
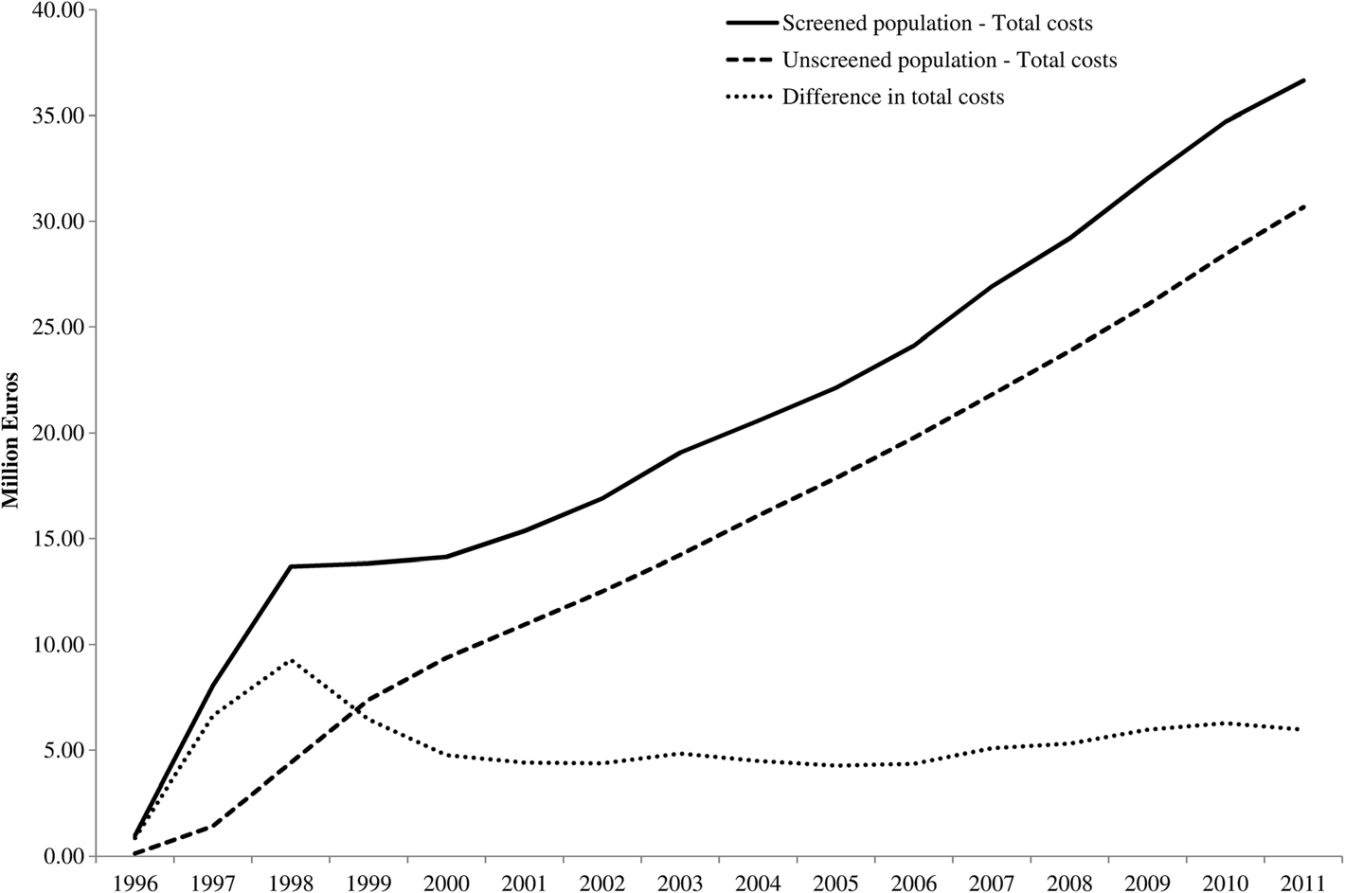
Short title: Budget impact analysis for the period from 1996 through 2011. Detailed legend: Budget impact analysis for the period from 1996 through 2011 for the scenarios with and without screening

Annual total costs for budget impact analysis are shown in Fig. 2. In 2011, more than 36 million euros were necessary to continue with the BCSPBC and the treatment costs related to previously detected BC; this estimation is growing yearly. As a consequence of the implementation of the screening programme, it had been necessary to add up to 9.2 million euros to the budget of the Basque Health Service in 1998. However, this figure became relatively stable from year 2000 onwards in annual 5.2 million euros.

## Discussion

The BC screening programme in the Basque Country proved cost-effective during the evaluation period with both multi-cohort and single-cohort approaches assuming the recommended threshold of 30,000€ per QALY [24]. When a 3 % discount was applied to costs and utilities from 2011 on, the ICER increased slightly but it was still far below the established threshold. The simultaneous use of a combined and a single-cohort approach was helpful to compare the efficiency of BC screening in real population dynamics (multi-cohort model) and incident cohort (single-cohort). In both cases, the results are valid only if the follow-up is long enough to achieve a steady state in the interaction between the natural history of BC and all its determinants that are modified by the screening. The steady state is defined as the time when each recently observed behaviour of the system (trade-off between short-term costs and long-term benefits) will remain constant in the future [25].

In a comparison of different screening programmes, De Koning pointed out the dependence of the cost-effectiveness on the attendance rate and the quality of the programme [5]. Thus, this ICER is within the range of the best programmes as the high participation rate (80 %) and other quality indicators of the Basque programme fit well the recommended guidelines [26, 27]. As noted in the literature, some of those favourable figures are related to the centralised system applied by the Basque Health Service to implement the BCSPBC [5]. Our results are similar to other studies carried out in the Spanish context that used ordinary, single-cohort cost-effectiveness analysis. Carles et al. obtained an ICER of 4,469€/QALY [28] in Catalonia. The MIcrosimulation SCreening ANalysis (MISCAN) model was developed in the 1980’s to evaluate the effects of breast cancer screening in the Netherlands [29] and applied to Navarra [30] resulted in an ICER of 2,650€/life-year gained (LYG), whereas, when the MISCAN model was applied to Catalonia, it resulted in 4,475€/LYG [31]. Interestingly, application of the MISCAN model in the Netherlands with the same strategy (women aged 50–70 invited every 2 years) resulted in a similar ICER (3,400€/QALY) [32].

Current guidelines for health economic evaluation and modelling have not adequately addressed the issue of cohort definition [33]. Although the standard approach is to use a single cohort, different authors have underlined the advantages of a multi-cohort method to reproduce real-world populations [7, 34]. Kuntz et al. [33] noted that if no substantial heterogeneity is found on the basis of characteristics of the screened women in the prevalent and incident cohorts, both approaches render similar results [33] and our results are in line with this affirmation. Similarly, O’Mahony et al. [12] highlighted how the ICER is influenced by the number of birth cohorts under differential discounting [34]. As we have used the same discounting, aggregating cohorts did not produce differences.

All investment decisions involve an opportunity cost, and therefore, a decision to spend on one option deprives the beneficiaries of another option [8]. Thus, investment in health care, curative and public health requires evidence of effectiveness and cost-effectiveness of competing interventions [35]. When we take into account both the 67.4 million euros invested in the screening programme during its first 15 years and the total cost of roughly 1,000 million euros (36 million euros in excess), it seems clear that an explicit statement is needed regarding the best use of those resources. Actually, due to the increase in BC incidence and longer survival times achieved by early detection, an increase in the prevalence of treated cancers occurred and thus, overall costs increased considerably. In addition, treatment costs would have continued, even if the screening programme had stopped in 2011. The complementary budget impact analysis showed how the overall annual costs varied in the first years of implementation and the difference between scenarios stabilized after 2000 at approximately five million euros. The small increase in 2007 is the result of the increased screening age of 70 years. The overall diagnosis and treatment cost of the BC for the women included in the programme in the Basque Country increased to 36.6 million euros in 2011.

The high attendance rate for the programme helped to reduce disparities in BC survival [36, 37]. Screening rejection has been proposed on the supposition that new cutting-edge treatments can offset the delay in diagnosis, thus, making it unnecessary to treat at an earlier stage [2]. This theory has not yet been confirmed, and, even if established, such an approach would not guarantee that innovative therapies would be available to all women with BC. On the contrary, high attendance rates in screening programmes means that the benefit now reaches every female subject in the programme without considering her socioeconomic level.

The retrospective nature of the design of this study posed some doubts about how to deal with discounting [8, 12, 17, 33]. Following the method of Stout et al, we discounted only the future costs and benefits [17]. In other words, the results (costs and QALYs) during the evaluation period (1996 to 2011) were directly aggregated, because they had already occurred, but we did discount the follow-up of women living after 2012 to their death as future costs and included QALYs. Although the ICER calculated without any discount changed from 4,214 to 2,294€ per QALY, the difference was not significant, because both figures were far below the usual threshold (30,000€/QALY). Similarly, from both single-cohort and multi-cohort models, we obtained almost the same ICER (4,600 and 4,200€/QALY), which underlines the efficiency of the programme.

The growing budget impact indicates that during these years women included in the programme progressively represented a larger portion of the treatment costs of BC. The more years of follow-up included in the programme, the closer the budget is to arriving at a plateau, as these figures include only screened women. These figures highlight that after 15 years of screening the difference between budgets in the two scenarios (screened and unscreened population) could still vary in the future.

## Conclusions

Our economic results confirm the epidemiological benefits related to the centralised screening system and support, first, the continuation of the programme and, second, the long follow-up required to fully evaluate the benefit of the programme. In terms of cost-effectiveness the ICER obtained in both population level evaluation and single-cohort assessment were far below the threshold used for decision making. However, in order to make the final decision it is necessary to take into account that five million Euros more were required annually in average in the budget of the Basque Health Services due to the implementation of the screening programme.

## Abbreviatons

BC, breast cancer; BCSPBC, breast cancer screening programme in the Basque Country; CI, confidence interval; ICER, incremental cost-effectiveness ratio; LYG, life years gained; MISCAN, microsimulation screening analysis; QALY, quality adjusted life years

## Additional file

Additional file 1: Model description. This file includes 465 the detailed description of the simulation model built for 4667 this study. (PDF 429 kb)
