# Supplementary material for: Economic evaluation of the breast cancer screening programme in the Basque Country: retrospective cost-effectiveness and budget impact analysis
Source: BMC Cancer. 2016 Jun 1;16:344. doi: 10.1186/s12885-016-2386-y (PMC4888560; doi:10.1186/s12885-016-2386-y)

COMPLETE MODEL DESCRIPTION

A simplified representation of the model constructed with Arena simulation software (Version 14.0, Rockwell software, Milwaukee, WI) is shown in Figure S1 and the data used to implement it is specified in Table S1.

Natural history

We modelled the natural history of BC using the approach adopted by Lee et al. (Lee and Zelen, 2006). Four main states of health were distinguished: (1) disease-free or undetectable BC; (2) asymptomatic BC that can be diagnosed by screening; (3) symptomatic BC diagnosed clinically; and (4) death from BC.

The age distribution was assigned based on the previous study by Rue et al. who estimated the functions for the onset of the preclinical phase. (Rue et al, 2009). They were based on BC incidence from Catalan cancer registries and a distribution of sojourn time in the pre-clinical state, those authors used a generalized linear model with a Poisson distribution and a polynomial parameterization for the variables of age and cohort for the estimation of BC incidence when no data was available (Rue et al, 2009). Upward breast cancer incidence trends were included in our model using cohort effects. We assumed that the sojourn time of the pre-clinical phase follows an exponential distribution as Lee et al. did based on results of clinical trials (Lee and Zelen, 2006). Specifically, Lee et al. used an exponential distribution with mean 4 years in the case of women aged 50 years or more; we calibrated this value according to observed age-specific BC incidence.

In this model we considered that every woman who reached the clinical state would be diagnosed clinically at the beginning of this state. Therefore we applied the age-specific distribution of BC detection-stages observed in the cancer registries of the Basque

Country in 1995, before the screening programme began for clinically detected BC (Table S2). In situ carcinomas were considered the lowest stage in which BC could be detected. On the basis of the work by Vilaprinyó et al. (Vilaprinyo et al, 2009), we applied distributions of age- and stage-specific survival in women diagnosed either clinically or by screening.

Mortality from causes other than BC was randomly assigned, depending on the woman's birth cohort, based on an empirical function. All-cause and BC-caused mortality data were obtained from the Basque mortality registry for the period 1986-2010 (Table S1). Data related to Basque women population by age and birth cohort were provided by The Basque Statistics institute (EUSTAT). In order to estimate the age at death from causes other than BC, by birth cohort, we used the actuarial method that removes breast cancer as a cause of death, described by Vilaprinyo et al. (Vilaprinyo et al, 2008). Thus each diagnosed woman was assigned two ages at death and the minimum of these two ages determined the cause and age of death.

Screening characteristics

The good quality of the programme data base allowed to calculate the exact number of women invited for the first time into the BCSPBC from 1996 through 2011, exactly 414,041 women (Table S3). Their age distribution was also obtained from the programme data base. From 1996 to 1998 during the programme implementation, the population consisted only of women invited for the first time, that is, cohorts aged 50 through 64 years. In subsequent years, instead, only cohort aged 50 to 51 years were invited for the first time. Actually, the target population also included several cohorts that had previously been invited to participate in the programme, apart from those that received the invitation for the first time. The extension of the target population from 50

to 64 years and then 50 to 69 years began in 2006, with women aged 65 years continuing in the programme until age 69 (Sarriugarte, 2011).

The total number of mammograms performed in the programme was determined by the number of invited women (including early recalls) and annual attendance rates, which were exactly known from the programme data base (Table S3). Annual attendance rates were considered independent as correlation of the participation in first and repeated screening rounds was not available.

Four phases were distinguished during the studied period due to the variability of sensitivity and specificity values and screen-detected BC stage distribution: (1) from 1996 to 1999, the implementation phase, when most of the women invited to the programme received their first invitation; (2) from 2000 to 2005, the prevalence phase, when the percentage of women invited for the first time was much lower than the percentage of women invited for successive mammograms; (3) from 2006 to 2008, extension phase, when the programme was extended to women aged 65 to 69 years; (4) from 2009 to 2011, digital phase, when the switch to digital mammography occurred. Observed screening mammography results were used together with the number of invited women and number of screening-detected breast cancers and observed interval cancers to calculate sensitivity and specificity for each of the defined phases (Table S4). In the model, a positive or negative screening result was assigned based on the woman's actual health status and the correspondent sensitivity and specificity of the programme. Observed data was also analysed to obtain the distributions of disease stages for screening-detected cases in the different phases of BCSPBC (implementation, prevalence, extension and digital) (Table S2). In addition, as two identical populations were created for the comparison of the screening and no-screening scenarios, the same random numbers were used to simulate the stage distribution for the clinically and the

screening-detected cancers in the same woman, in order to estimate the advance in detection stage due to screening.

Model calibration and validation

The model was run in the screened scenario for the whole female population invited at least once into the BCSPBC during the study period in order to reproduce the actual performance of the programme.

Three main parameters were calibrated: time between consecutive invitations, age distribution of preclinical phase onset and its mean duration. We obtained the best fitting parameters to include in the final model by following the seven-step approach for calibrating models by Karnon et al. (Karnon and Vanni, 2011).

Random search and grid search algorithms were combined, and 25 simulations were run for each possible value. The goodness-of-fit measure applied to assess the difference between observed and estimated outcomes was the chi-square statistic. The overall chi-square statistic of each hypothesis was calculated as the sum of the chi-square statistics calculated for the analysed years. We assumed outcomes for each year to be independent and uncorrelated. Finally, we included in the model the parameter value for which the overall chi-square was the lowest.

First, we calibrated the time between intervals considering that it was not influenced by other unobserved parameters. At the beginning, we used a random search algorithm considering different values from a normal distribution centred in 2 years and standard deviation 0.5. Based on these results, we continued using a grid search algorithm, running 25 simulations for 10 different values between 2.11 and 2.20. The goodness-of-

fit measure applied to assess the difference between observed and expected outcomes was the chi-square statistic. We included in the model the parameter value for which the overall chi-square statistic was the minimum: 2.18 year between consecutive invitations (Figure S2).

Afterwards, we calibrated jointly two factors. The first one will be the relative risk (RR) for the incidence function. The second multiplier will be used to calibrate the mean value for the preclinical state duration which prior estimate was 4.0. Thus we will calibrate the factor t to obtain a final mean preclinical state duration 4t. We considered as target outputs the number of screening-detected cancers from 1996-2011, together with total cancer detection rates by age group (50-54, 55-59, 60-64, 65-69) for the period 1999-2009. Random search algorithm was used also in this case considering Normal(1,0.25) distribution for both parameters for a first approximation and a grid search algorithm centred in $0.87 \leq RR \leq 0.90$ and $0.85 \leq t \leq 0.90$. The goodness-of-fit measure used in this case was also the chi-square statistic. The final relative risk used for BC incidence functions was 0.88, and the mean time in preclinical state 3.44 years (Figure S3).

For model validation, we compared the estimated results for the screened population (multi-cohort model) with the observed indicators from BCSPBC and the Basque cancer registries such as number of invited women (Figure S2), number of mammograms carried out in the programme (Figure S2), age-specific breast cancer incidence (Figure S3) or the number of women with a positive mammography result (Figure S4). We also confirmed that life expectancy for women from the general population and women who died from BC was concordant with the observed data (Table S5).

Probabilistic Sensitivity Analysis

The probabilistic feature of the model is based on varying the main variables randomly at the same time [21]. Each variable has assigned a distribution fitting the range of all possible values and at the beginning of each simulation a random generator selects the value for each variable from the specified distribution. This permits to examine the effect of joint uncertainty in the variables of the model. The distributions used for the main parameters varied in the probabilistic sensitivity analysis were detailed in Table S6.

Time between invitations was calibrated with the aim of reproducing the number of invitations carried out in the programme and the optimal value obtained was 2.18 years. Therefore a uniform distribution was used for this parameter centred in 2.18 and including the theoretical value 2.00 years. The same occurred for the mean value of the duration of the pre-clinical state, where a uniform distribution centred in 3.44, calibrated value, and including 4.00, theoretical value, was used.

On the other hand, a Beta distribution was used both for sensitivity and specificity values. In this case the parameters were based on the number of cases observed in the screening programme in the period 1996-2011: true positive and false negative results for sensitivity and true negative and false positive results for specificity.

Finally, Dirichlet distribution was used for the distribution of detection-stage on screen-detected cancers. The parameters used for Dirichlet are mainly the number of cases observed in the screening programme for each detection-stage depending on the period and detection-age.

The cost-effectiveness plane displays the incremental cost (vertical axis) and effectiveness (horizontal axis) results of 1,000 simulation runs. In addition, the

acceptability curve represents the probability that breast cancer screening is cost-effective compared with no screening for varying threshold values of the cost-effectiveness ratio [21] (Figure S5). The ICER obtained in each of the 1,000 runs is confronted with the different thresholds to calculate those probabilities.

Variability in participation rates was not included in the main probabilistic sensitivity analysis as the observed values were obtained from a sample of about 115,000 women each year the variability was assumed very small. However, as we were concerned about the interest on the variation of this parameter we ran the main single-cohort model for two more scenarios with lower participation rates: 50% and 30%.

BIBLIOGRAPHY:

Izarzugaza MI, Martinez R, Audicana C, Larrañaga N, Hernandez E, Tobalina MC, De la Cruz M, Laviñeta M, Hurtado R, San Sebastian MC, De Miguel AR, Michelena MJ, Lopez de la Calle J. Cancer in the Basque Country: Incidence, mortality, survival and their trends. Vitoria-Gasteiz: *Department of Health and Consumer Affairs, Basque Country Administration;* 2010.

Karnon J, Vanni T (2011) Calibrating models in economic evaluation. *Pharmacoeconomics* **29**: 51-62.

Lee S, Zelen M (2006) A stochastic model for predicting the mortality of breast cancer. *J Natl Cancer Inst Monogr*: 79-86.

Rue M, Vilaprinyo E, Lee S, Martinez-Alonso M, Carles MD, Marcos-Gragera R, Pla R, Espinas JA (2009) Effectiveness of early detection on breast cancer mortality reduction in Catalonia (Spain). *BMC Cancer* **9**: 326-335.

Sarriugarte G (2011) *Generalidades del Programa de Detección Precoz de Cáncer de Mama del País Vasco* [Online]. Departamento de Salud del Gobierno Vasco. 2011. Available in: http://www.osakidetza.euskadi.net/r85-ckenfe11/es/contenidos/informacion/cancer_mama/es_canc_mam/generalidades.html

Vilaprinyo E, Gispert R, Martinez-Alonso M, Carles M, Pla R, Espinas JA, Rue M (2008) Competing risks to breast cancer mortality in Catalonia. *BMC Cancer* **8**: 331-338.

Vilaprinyo E, Rue M, Marcos-Gragera R, Martinez-Alonso M (2009) Estimation of age- and stage-specific Catalan breast cancer survival functions using US and Catalan survival data. *BMC Cancer* **9**: 98-111.

Table S1: Model input and validation parameters

Input data	Source
Invited population	
Number of women invited for the first time	Screening programme data
Age distribution	Screening programme data
Participation rate	Screening programme data
Time until event	
Other cause mortality	Basque mortality registry
Breast cancer mortality	Basque mortality registry
Time till pre-clinical state	Rue et al, 2009
Pre-clinical state duration	Lee and Zelen, 2006
Age- and stage-specific breast cancer survival	Vilaprinyo et al.
Detection data	
Clinically detected cancer stage distribution	Basque cancer registry
Programme sensitivity and specificity	Screening programme data
Screen detected cancer stage distribution	Screening programme data

Validation data	Source
Invited population	
Total number of invited women	Screening programme data
Total number of mammograms	Screening programme data
Recall rate	Screening programme data
Remitted for additional test	Screening programme data
Detection data	
Age- and year-specific breast cancer incidence	Basque cancer registry
Screening-detected cancers	Screening programme data

Table S2: Distribution of breast cancer detection stages.

Detection stage	In situ	Stage I	Stage IIa	Stage IIb	Stage III	Stage IV
Clinically detected cancer						
50-59	24	78	59	38	22	19
	10.00 %	32.63 %	24.75 %	15.75 %	9.00 %	7.88 %
60-69	17	50	52	60	31	18
	7.42 %	21.72 %	22.86 %	26.29 %	13.72 %	8.00 %
>69	9	25	58	25	50	40
	4.35 %	12.11 %	27.85 %	12.11 %	24.22 %	19.37 %
Screen detected cancer	In situ	Stage I	Stage IIa	Stage IIb	Stage III	Stage IV
Period 1996-1999						
50-59	101	255	99	38	16	2
	19.69 %	49.71 %	19.30 %	7.60 %	3.12 %	0.58 %
60-69	87	247	93	32	19	4
	17.94 %	50.93 %	19.18 %	6.80 %	4.12 %	1.03 %
Period 2000-2005						
50-59	323	846	350	109	80	12
	18.77 %	49.16 %	20.34 %	6.39 %	4.65 %	0.70 %
60-69	190	600	163	56	36	3
	18.08 %	57.18 %	15.60 %	5.33 %	3.43 %	0.38 %
Period 2006-2008						
50-59	323	846	350	109	80	12
	18.77 %	49.16 %	20.34 %	6.39 %	4.65 %	0.70 %
60-69	190	600	163	56	36	3
	18.08 %	57.18 %	15.60 %	5.33 %	3.43 %	0.38 %
Period 2009-2011						
50-59	124	326	139	32	30	7
	18.76 %	49.47 %	21.18 %	4.99 %	4.54 %	1.06 %
60-69	95	332	122	33	24	2
	15.55 %	54.50 %	19.97 %	5.56 %	3.93 %	0.49 %

Table S3: Number of women invited into the breast cancer screening programme in the Basque Country and participation rates (%).

	First invitations		Successive invitation	
Year	Number of women	Participation	Number of women	Participation
1996	7,835	79.71	0	-
1997	67,719	72.94	0	-
1998	87,967	78.26	16,702	71.49
1999	41,841	84.60	51,037	64.57
2000	17,426	96.27	80,399	74.77
2001	18,902	90.45	86,792	70.82
2002	16,401	90.04	86,110	74.54
2003	21,109	84.38	87,877	74.59
2004	16,363	87.26	86,327	75.08
2005	14,043	89.49	91,996	75.35
2006	16,804	86.39	114,691	73.97
2007	17,018	87.92	105,850	75.18
2008	17,847	83.85	110,542	75.15
2009	18,510	85.68	116,330	75.51
2010	17,711	88.48	120,481	79.45
2011	16,545	91.21	128,836	79.49

Table S4: Sensitivity and specificity of the breast cancer screening programme.

Year	1996-1999	2000-2005	2006-2008	2009-2011
Sensitivity	95.20	83.40	83.52	85.86
Specificity	90.44	90.61	93.67	94.13

Table S5: Validation of the mean life expectancy for women in the general population and median survival time corrected by lead time for women with death from BC.

	Theoretical	Estimated
General population	83.70	82.61
BC death survival*		
Stage I	9.03	6.34
Stage IIa	6.46	4.77
Stage IIb	5.14	4.19
Stage III	3.41	2.74
Stage IV	0.80	0.63

*Median BC survival times when no other cause deaths occur are shown as theoretical. Estimated median survival times for BC deaths are lower than theoretical as women with greater BC survival time die from other causes.

BC = breast cancer

Table S6: Parameters uncertainty included in the probabilistic sensitivity analysis.

Variable	Distribution
Time between invitations	Uniform (2.00, 2.36)
Sensitivity	Beta (5261, 850)
Specificity	Beta (1210790, 100650)
Preclinical state duration	Uniform (2.88, 4.00)
Screen-detected cancer stage	Dirichlet (Table S2)

Figure S1: Simplified diagram of the model

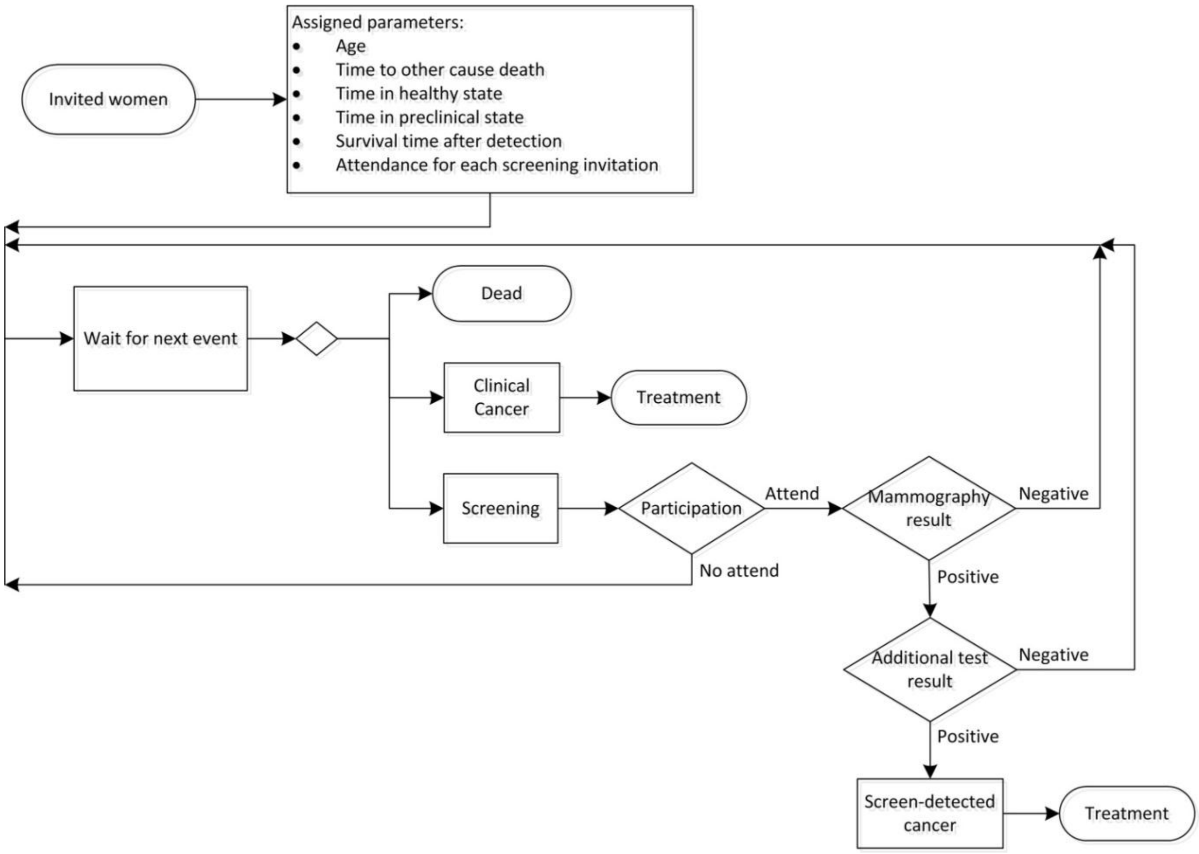

Figure S2: Total number of women invited to join the programme and the number of the mammograms carried out.

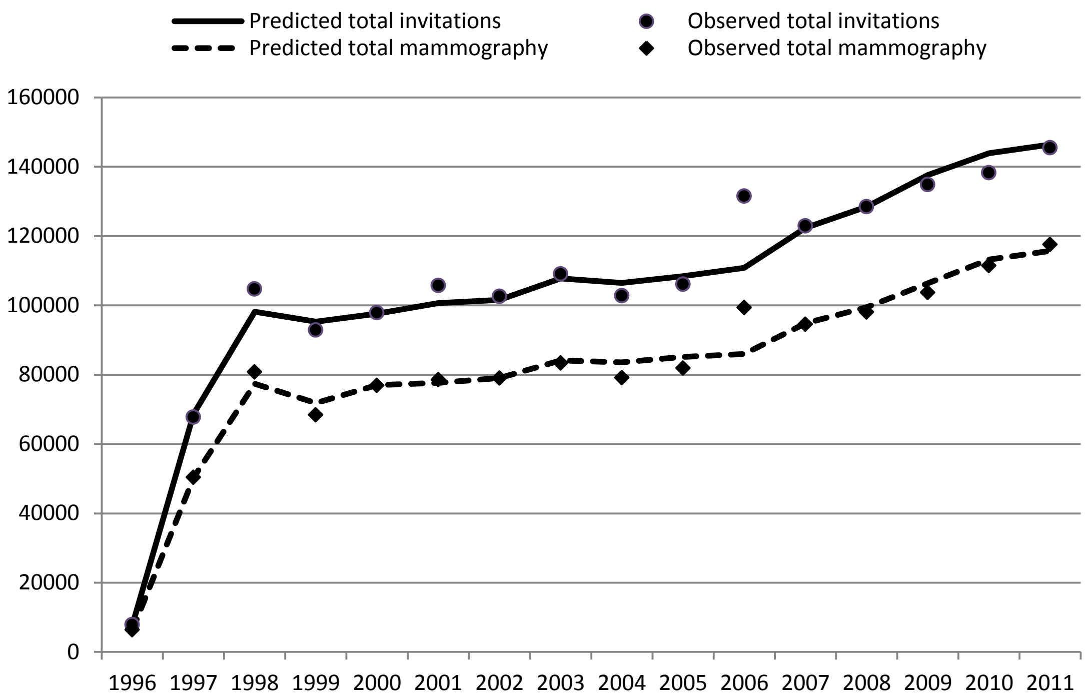

Figure S3: Breast cancer incidence by age group (implementation period 1996-1999 excluded).

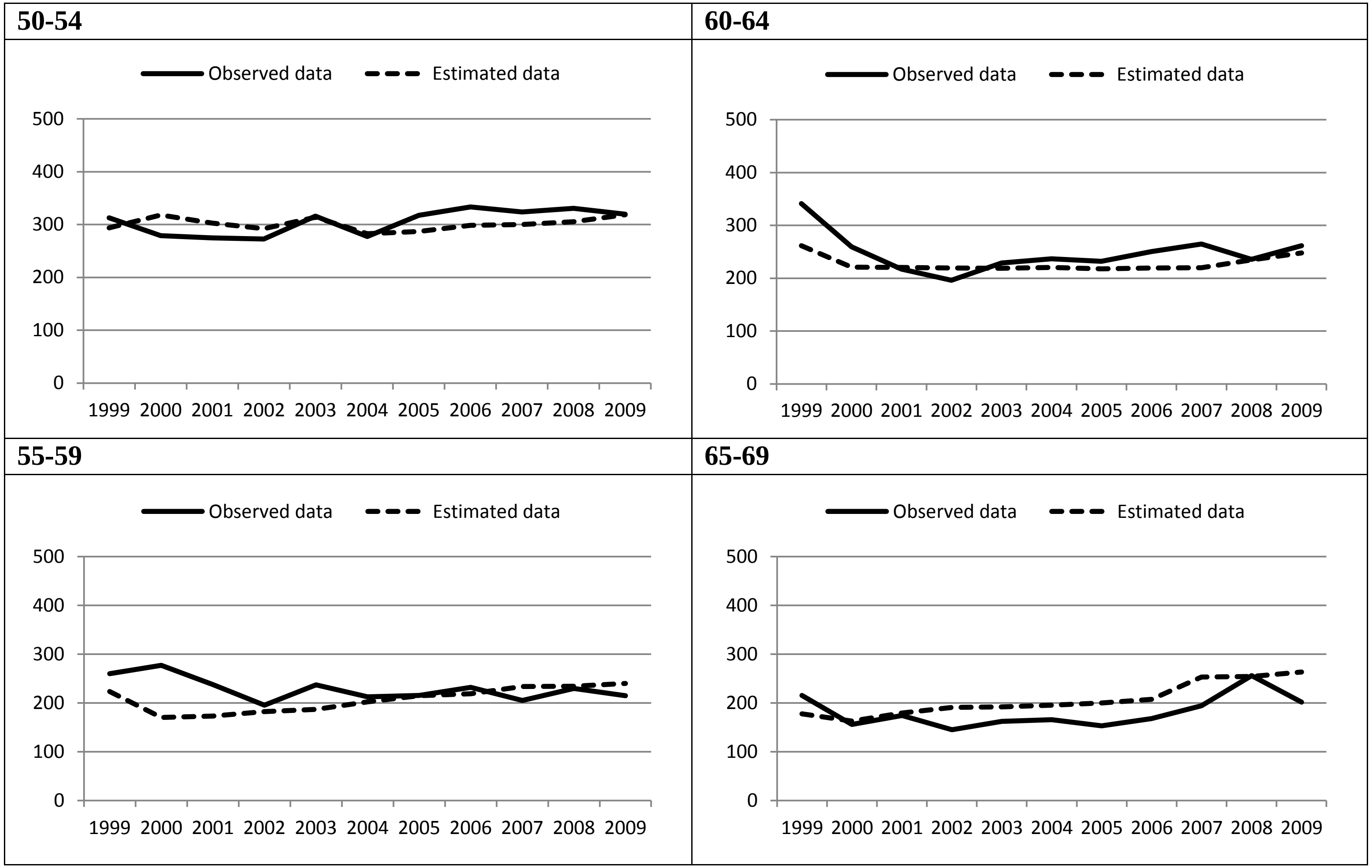

Figure S4: Total number of positive mammogram results in the breast cancer screening programme.

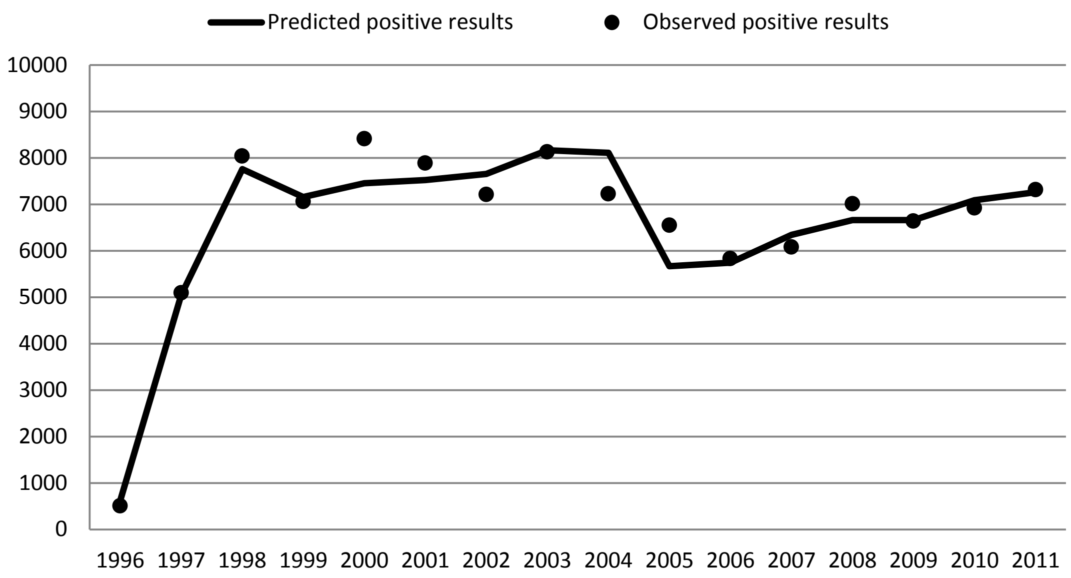

Figure S5: Acceptability curve related to the probabilistic sensitivity analysis in the multi-cohort model.

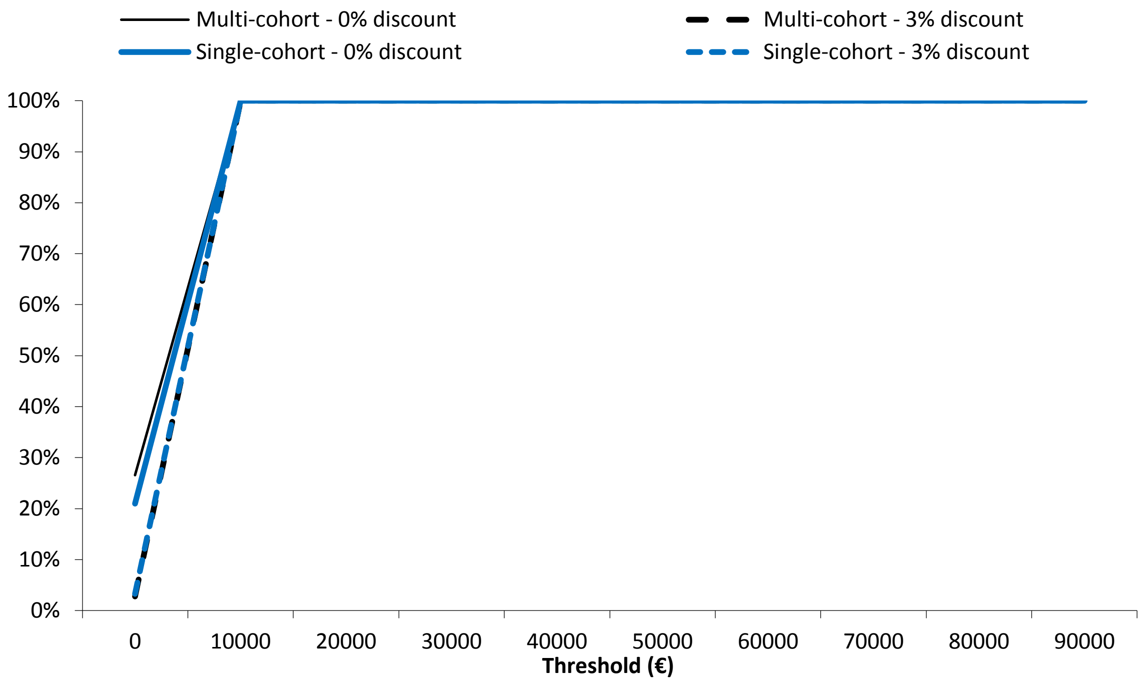

Supplement: Additional file 1: — Model description. This file includes 465 the detailed description of the simulation model built for 4667 this study. (PDF 429 kb) [file 12885_2016_2386_MOESM1_ESM.pdf]
